# Apoptosis Governs the Elimination of *Schistosoma japonicum* from the Non-Permissive Host *Microtus fortis*

**DOI:** 10.1371/journal.pone.0021109

**Published:** 2011-06-22

**Authors:** Jinbiao Peng, Geoffrey N. Gobert, Yang Hong, Weibin Jiang, Hongxiao Han, Donald P. McManus, Xinzhi Wang, Jinming Liu, Zhiqiang Fu, Yaojun Shi, Jiaojiao Lin

**Affiliations:** 1 Shanghai Veterinary Research Institute, Chinese Academy of Agricultural Sciences, Key Laboratory of Animal Parasitology, Ministry of Agriculture, Shanghai, China; 2 Queensland Institute of Medical Research, Herston, Queensland, Australia; University of Rochester, United States of America

## Abstract

The reed vole, *Microtus fortis*, is the only known mammalian host in which schistosomes of *Schistosoma japonicum* are unable to mature and cause significant pathogenesis. However, little is known about how *Schistosoma japonicum* maturation (and, therefore, the development of schistosomiasis) is prevented in *M. fortis*. In the present study, the ultrastructure of 10 days post infection schistosomula from BALB/c mice and *M. fortis* were first compared using scanning electron microscopy and transmission electron microscopy. Electron microscopic investigations showed growth retardation and ultrastructural differences in the tegument and sub-tegumental tissues as well as in the parenchymal cells of schistosomula from *M. fortis* compared with those in BALB/c mice. Then, microarray analysis revealed significant differential expression between the schistosomula from the two rodents, with 3,293 down-regulated (by ≥2-fold) and 71 up-regulated (≥2 fold) genes in schistosomula from the former. The up-regulated genes included a proliferation-related gene encoding granulin (*Grn*) and tropomyosin. Genes that were down-regulated in schistosomula from *M. fortis* included apoptosis-inhibited genes encoding a baculoviral IAP repeat-containing protein (SjIAP) and cytokine-induced apoptosis inhibitor (SjCIAP), genes encoding molecules involved in insulin metabolism, long-chain fatty acid metabolism, signal transduction, the transforming growth factor (TGF) pathway, the Wnt pathway and in development. TUNEL (terminal deoxynucleotidyl transferase dUTP nick end labeling) and PI/Annexin V-FITC assays, caspase 3/7 activity analysis, and flow cytometry revealed that the percentages of early apoptotic and late apoptotic and/or necrotic cells, as well as the level of caspase activity, in schistosomula from *M. fortis* were all significantly higher than in those from BALB/c mice.

## Introduction

Schistosomiasis is one of the most widespread and prevalent parasitic diseases of clinical and public health relevance. More than 46 species of mammals have been reported to be naturally infected with the causative agent in China, *Schistosoma japonicum*. The reed vole, *Microtus fortis*, is the only mammal in which the growth, development and maturation of this schistosome is prevented, resulting in the failure of the parasite to mature and complete its life cycle [1]–[3]. Previous investigations have suggested that humoral and/or cellular immunity may play an important role in the restricted development of *S. japonicum* in *M. fortis*. However, less attention has focused on other factors such as apoptosis, nutrition, neuroendocrine hormones, signalling pathways and other environmental parameters that might affect parasite survival and development in this host [4], [5]. BALB/c mouse is a susceptible host for S. japonicum and nearly 70% of *S. japonicum* in BALB/c mice can complete the lifecycle. Comparing differences in the molecular profiles of schistosomula from susceptible and non-permissive host species can provide important insights associated with schistosome development and, more specifically, the nature of the anti-schistosomal mechanism that occurs within *M. fortis*. The analysis may identify key molecules which are essential for the survival and development of the schistosome, as well as providing a novel approach for defining potential vaccine candidates or drug targets for the control of schistosomiasis [6].

Apoptosis is a tightly regulated process by which cells establish an inducible non-necrotic cellular death process, and it has a major role in balancing cell proliferation and remodeling tissue activity in many organisms. The mechanism for apoptosis in multicellular organisms includes the activation of caspases via mitochondrial-mediated mechanisms and death receptor-mediated mechanisms, resulting in apoptotic DNA fragmentation, nuclear chromatin condensation and the formation of apoptotic bodies and a distinctive apoptotic cellular phenotype [7]. Genomic studies have suggested that schistosomes have an apoptotic pathway that is similar to that of higher organisms [8]. A better understanding of the molecular and cellular mechanisms of schistosome apoptosis could provide new insights into the pathogenesis of schistosomiasis and might also lead to the development of new approaches for disease control. However, little work has been done to investigate the degree of apoptosis occurring in schistosomula, or in the anti-schistosomiasis animal model, *M. fortis* [9].

In recent years, *M. fortis* has been used as a non-permissive animal model to investigate which molecular targets may be involved in the interactions between the schistosome parasite and its host. Several possible molecules were identified by screening a *S. japonicum* cDNA library with cellular preparations from host lungs and liver as well as sera from *M. fortis* [10], [11]. In addition, microarray techniques have been used for gender, strain-, developmental and tissue-specific profiling of the schistosomes and have provided valuable new information on schistosome biology [12], [13].

In the current study, ultrastructural, transcriptional and biochemical differences between schistosomula isolated from *M. fortis* and BALB/c mice were compared using scanning electron and transmission electron microscopy, MTT (3-(4,5-dimethylthiazol-2-yl)-2,5-diphenyltetrazo -lium bromide) examination, terminal deoxynucleotidyl transferase dUTP nick end labeling (TUNEL) assays, caspase 3/7 activity analysis, flow cytometry (FCM) and microarray analysis. The aims of the study were to further understand schistosome development, the nature of the schistosome development and failure mechanism in *M. fortis*, and to identify differentially expressed genes that might play a role in the developmental biology and survival of *S. japonicum*.

## Materials and Methods

### Host animals and parasites

All animal care and experimental procedures were conducted according to the guidelines for animal use in toxicology. The study protocol was approved by the Animal Care and Use Committee of the Shanghai Veterinary Research Institute, Chinese Academy of Agricultural Sciences (CAAS). The use of experimental animals in this study was approved under Project A001 by the Animal Ethics Committee of the Shanghai Veterinary Research Institute, Chinese Academy of Agricultural Sciences (CAAS).

Eight-week-old specific-pathogen free (SPF) female *M. fortis* (∼60 g) and BALB/c mice (∼20 g) were purchased from *Xipu'er-bikai* Experimental Animal Co., Ltd (Shanghai) and Shanghai Laboratory Animal Center, Chinese Academy of Sciences (Shanghai), respectively. The life cycle of *S. japonicum* (Chinese mainland strain, Anhui isolate) was maintained in New Zealand rabbits and *Oncomelania hupensis* snails at the Shanghai Veterinary Research Institute, CAAS. *Microtus fortis* and BALB/c mice were infected with 3,000 and 200 cercariae, respectively. Infected animals were perfused by phosphate buffered saline (PBS) at 37°C at 10-days post infection and the schistosomula were collected. Special attention was paid to removing all host tissue from the isolated parasites through multiple washings with phosphate-buffered saline (PBS) (pH 7.4) at 37°C.

### Microscopy observations

The length and width of individual schistosomula obtained from *M. fortis* and BALB/c mice (30 worms from each host species) were measured using light microscopy. *M. fortis* and BALB/c mice (each group with 30 animals) were perfused to calculate the survival rate of *S. japonicum* worms. For SEM observations, schistosomula from the two host species were fixed with 2.5% glutaraldehyde/PBS (pH 7.4) at 4–8°C, post-fixed with 1% (w/v) osmium tetroxide, and then with isopentyl acetate for 1.5 h before being dehydrated in an ascending ethanol series. The samples were critical-point dried, and then coated with ∼200A of gold in an ion coater before SEM observations were performed, using a JEOL6380LV microscope.

For TEM observations purified schistosomula were fixed in 2.5% glutaraldehyde/PBS (pH 7.4) at 4–8°C, washed with PBS, post-fixed with 1% osmium tetroxide, washed with distilled water and then dehydrated using dimethylketone. After embedding in araldite resin, 50 nm-thick sections were processed and stained with uranyl acetate and lead citrate, and then examined using a HITACHI, H-600TEM.

### Total RNA isolation, hybridization and feature extraction

Total RNA was isolated from schistosomula using TRIzol Reagent (Invitrogen, USA) and quantitated by ultraviolet spectrometry (Eppendorf Biophotometer) and a Nano-Drop ND-1000 spectrophotometer (Thermo Scientific, USA). The quality of total RNA was assessed using a Bioanalyser RNA Pico Lab Chip (Agilent) before storage at −80°C.

The microarray used in the study was constructed by the Agilent Technologies Company based on the transcriptome of *S. japonicum* according to a protocol described elsewhere [14]. The microarray design resulted in 14,171 target contiguous sequences (contigs) printed three times, plus proprietary positive and negative controls (Agilent Technologies). Contigs were based on the nucleotide sequences associated with a recent proteomics publication [15]. Microarrays were printed in a 4×44 k feature format. Full details of this schistosome microarray design have been deposited in the GEO (Gene Expression Omnibus, http://www.ncbi.nlm.nih.gov/geo/) public database with an associated platform accession no. GPL9759. The microarray data from the current study have also been deposited in GEO with the series accession no. GSE25728. The entire microarray data is MIAME compliant in this research.

A 300-ng aliquot of total RNA was used to synthesize fluorophore-labelled cRNA using cyanine 3-CTP (CY3c) as described (Agilent Technologies: One-Color Microarray-Based Gene Expression Analysis). Samples were examined at A260 and A550 using a ND-1000 spectrophotometer to determine yield, concentration, amplification efficiency and abundance of CY3c. Microarray hybridizations were performed in duplicate for all samples.

### Data analysis, gene ontology and KEGG pathway analysis

Microarray slides were scanned using an Agilent Microarray Scanner (B version) at 550 nm. The ‘tag image format files’ (Tiffs) processed with the scanner were loaded into the Feature Extraction 9.5.3.1 image analysis program (Agilent) to produce standardized data for statistical analysis. All microarray slides were assessed for background evenness by viewing the tiff image by Feature Extraction. Feature-extracted data were analyzed using GENESPRING (version 7.3.1; Agilent Technologies/Silicon Genetics, Redwood City, CA, USA). Microarray data were normalized using a normalization scenario for ‘Agilent FE one-color’, which including ‘Data Transformation: Set measurements less than 5.0 to 5.0’, ‘Per Chip: Normalize to 50th percentile’ and ‘Per Gene: Normalize to median’.

Data sets were further analyzed using published procedures based on one-color experiments and utilized gProcessedSignal values determined from Agilent's Feature Extraction software and GeneSpring microarray software (Agilent Technologies Version 10), including aspects of the signal:noise ratio, spot morphology and homogeneity. ProcessedSignal represents the signal after localized background subtraction and includes corrections for surface trends. Features were deemed ‘Absent’ when the processed signal intensity was less than twice the value of the processed signal error value. Features were deemed ‘Marginal’ when the measured intensity was at a saturated value or if there was a substantial amount of variation in the signal intensity within the pixels of a particular feature. Features that were neither absent nor marginal were deemed ‘Present’. Data points were included only if they were present or present/absent and probes or contigs were retained if all data points were present or present/absent.

Protein blast and gene ontology analysis using Blast2Go Batch BlastX (six-frame translation protein homology) was performed at http://www.blast2go.de on all contigs. Gene ontology correlations with relative gene expression values were made using ErmineJ software. In addition, the Kyoto Encyclopedia of Genes and Genomes (KEGG) pathway of the differential expression genes were analyzed by using the maps available at http://www.genome.jp/kegg/.

### Validation of microarray data with qPCR analysis

Ten identified differentially expressed genes from *M. fortis* schistosomula were chosen for validation of the microarray results using qPCR. Forward and reverse primers (Invitrogen, Shanghai, China) were designed according to the sequences of the eight contigs tested. Total RNAs of the schistosomula harvested from *M. fortis* and from BALB/c mice were isolated using TRIzol® Reagent (Invitrogen, USA) following the manufacturer's instructions, quantified by ultraviolet spectrometry (Eppendorf Biophotometer), and used for reverse transcription using a SuperScript™ II Reverse Transcriptase kit (Invitrogen, USA) and pd (N)6 random hexamer primers. The relative expression quality of schistosomula from the two different hosts was validated by quantitative qPCR using the RG-3000A qPCR system (RoterGene, USA) and SYBR® Premix Ex Taq™ (Perfect Real Time) kit (Takara, China).The qPCR reaction mixture (20 µl) contained 10 µl SYBR® Premix Ex Taq™ (2X), 0.2 µl of forward and reverse primers mixtures, 1 µl cDNA template, and 7 µl RNase-free distilled H_2_O. Reaction conditions were as described in the SYBR green kit and the cycling protocol was as follows: 95°C for 10 s and 40 cycles of 95°C for 5 s, 55°C for 10 s and 72°C for 15 s, acquiring fluorescence at the end of each extension step; three repeats were carried out for each sample. The PCR products were detected in real time using the Rotor-Gene 3000A Dual Channel Multiplexing System. The gene encoding NADH-ubiquinone reductase was selected as a housekeeping gene to normalize the expression differences in schistosomula from *M. fortis* and from BALB/c mice.

### Analysis of the apoptosis-associated genes with qPCR

Further analyses of the apoptosis-associated genes in schistosomula from *M. fortis* 10 days post infection were carried out using the qPCR technique. The apoptosis-associated genes included *Sj* caspase 3(Contig00177), *Sj* caspase 7(Contig06377) and cytokine-induced apoptosis inhibitor (CIAP) (Contig04500), selected following the analysis of differential gene expression from the microarray data. Three additional apoptosis-associated genes [encoding apoptosis inducing factor (AIF), Bcl-2, *Sj* caspase 9], not revealed in the microarray analysis, were found in the apoptosis pathway described in the Genome Pathway Mapping System of the *S. japonicum* genome project database (http://lifecenter.sgst.cn/schistosoma/cn/ genomeProject.do).

### Cellular proliferation and apoptosis assays of schistosomula cells

Schistosomula obtained from the two host species were washed three times in PBS by careful centrifugation (1000×g, 5 min). Schistosomula cells were harvested with the methods reported as reported [16].

The worms were washed three times with 0.25% trypsin and 0.02% EDTA (trypsin and EDTA were all dissolved in the calcium- and magnesium-free phosphate-buffered saline (CMF-PBS)). After 15 min setting, the worms were minced in 0.2 ml of the trypsin/EDTA mixture solution and then the trypsin/EDTA mixture solution was added to 10 ml to digest for nearly 4 h at 4 °C with timely shaken, and then the solution was homogenized with a Pasteur pipette for 30 min. The suspension containing cells and fraction suitable for culture was transferred to another tube. The underlayer tissue, containing unfragmented worms and large fragments, was re-homogenized completely with a Pasteur pipette. After centrifugation at 1200 rpm for 15 min, the sedimentary cell pellet was resuspended in culture media and then homogenized and filtered with a Cell Strainer (40 µm SPL, Shanghai, China) to isolate single-cell preparations of schistosomula.

The proliferation of cells on schistosomula was assessed using a MTT cell viability/cytotoxicity assay kit (Beyotime Biotechnology, China), which quantifies the ability of viable cells to convert soluble MTT dye into an insoluble dark-blue formazan reaction product. MTT stock solution was added (as a 1:10 dilution) to cells in each well of a 96-well tissue culture plate, which was then incubated at 37°C for 4 h. The medium containing MTT was then removed and 150 µL dimethylsulfoxide (DMSO) was added per well to dissolve the formazan crystals. The absorbance in each well was measured at 570 nm using a Synergy HT Multidetection Reader (Bio-Tek Instruments, USA). Cultures without cells were used as blank controls.

The Annexin V-FITC/propidium iodide (PI) apoptosis detection kit (Promega, USA) was used according to the manufacturer's protocol. The analysis was performed by flow cytometry (FCM) (Becton, Dickinson and Company, NJ, USA). Approximately 10,000 schistosomula cells were tested for each sample, and the experiment was repeated three times.

### DeadEnd™ Fluorometric TUNEL analysis of schistosomula cells

The TUNEL system was used to assay apoptotic cell death by measuring nuclear DNA fragmentation with the fluorescein-12-dUTP, which is an important biochemical hallmark of apoptosis. The fluorescein-12-dUTP labeled DNA can be visualized directly by fluorescence microscopy on directly staining schistosomula and on schistosomula cells by flow cytometry.

Schistosomula cells for DeadEnd™ Fluorometric TUNEL analysis were prepared according to the Suspension Cell Apoptosis Analysis instructions in the manufacturer's handbook (DeadEnd™ Fluorometric TUNEL System, Promega, Wisconsin, USA). The analysis was performed by FCM (Becton, Dickinson and Company). Approximately 10,000 schistosomula cells were evaluated for each sample, and the experiment was repeated for 3 times.

### Caspase activity analysis

Schistosomula were lysed with lysis buffer containing an EDTA-free inhibitor (Roche, Switzerland), and the mixture washed by centrifugation at 3000×g for 5 min. Caspase activity in the schistosomula lysates was measured using a Glo™ 3/7 Assay kit (Promega) according to the manufacturer's instructions. 80 µl of a caspase Glo 3/7 reagent was added to each well of the schistosomula lysates in 24-well plates, and the contents of the wells were mixed gently using a plate shaker at 400 rpm for 30 s. The plates were incubated at room temperature in the dark for 90 min to allow the reaction to occur. 100 µl of reaction solution was then measured with a luminometer (Berthold, Germany). A negative control was performed using 100 µl of lysis buffer alone. The protein concentration of each reaction solution was assessed using a Pierce BCA protein assay kit and Compat-Able protein assay preparation reagent set (Pierce, USA) for normalizing luciferase.

### Statistical analysis

Data were expressed as the mean ± SD. Statistical analyses were performed using the Student's *t* test. Values of p<0.05 were considered to be significant. The analysis of correlation between microarray and quantitative PCR was performed in GraphPad Prism Version 5 (Graphpad Software Inc.) and was based on a previously published analysis [17]. To correlate results from the microarray and quantitative PCR platforms, we first determined whether the data were distributed normally. This involved the use of both the “D'Agostino & Pearson omnibus normality test” and “Shapiro-Wilk normality test”. Both tests indicated that the data were not normally distributed; thus a Spearman correlation (Rho) was employed. All methods used an alpha value of 0.05.

## Results

### The general growth and survival rates of schistosomula

Schistosomula were collected from *M. fortis* and BALB/c mice 10 days post infection. The length, width and survival rate of schistosomula were measured by light microscopy and the differences are described as follows. Compared with the average length and width of schistosomula from BALB/c mice (length: 878.50±137.45 µm; width: 159.10±47.37 µm), the length and width of schistosomula from *M. fortis* were significantly (*p*<0.01) decreased (length: 402.20±102.26 µm; width: 88.30±20.03 µm) with clear retardation in growth. The survival rate of 10 day post infection schistosomula from *M. fortis* was significantly lower, being ∼10% compared with ∼73% of that for the schistosomula from the BALB/c mice.

### Ultrastructural features of schistosomula

**Figure 1:**
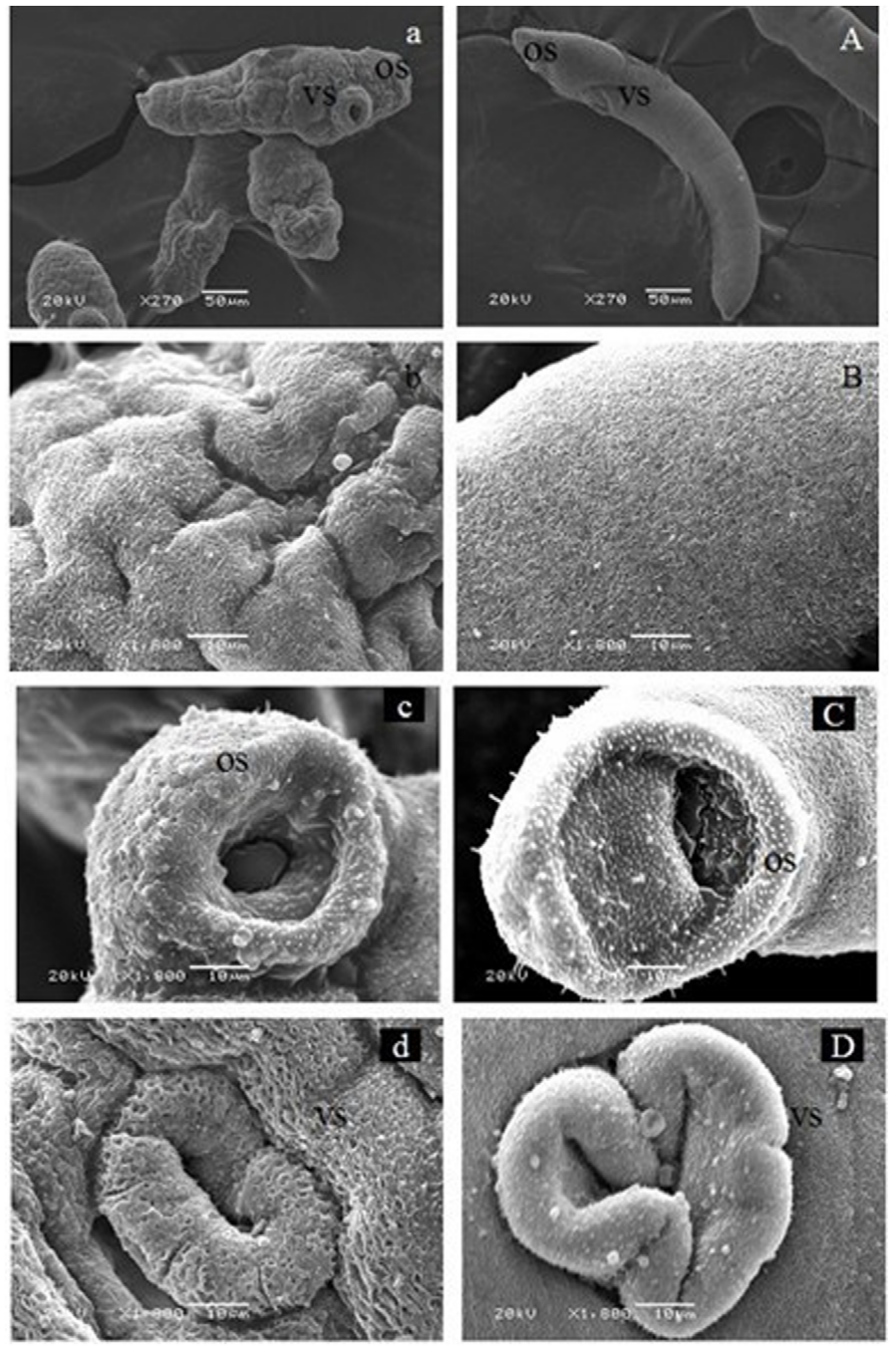
Scanning electron microscopy (SEM) of schistosomula from *Microtus fortis* (a,b,c,d) and BALB/c mice (A, B, C, and D). Os, oral sucker; Vs, ventral sucker.

SEM observations revealed significant differences in the surface topography of schistosomula from BALB/c mice (Figure 1A–D) compared with those from *M. fortis* (Figure 1a–d). For example, the schistosomula from *M. fortis* had a withered blebby appearance, with a vacuole in the oral sucker and a flabby ventral sucker. In addition, tegument sloughing was apparent, which resulted in lysis of the tegumental matrix. In general, the ultrastructure was similar in appearance to that reported for lung schistosomula of *S. japonicum* [18].

**Figure 2:**
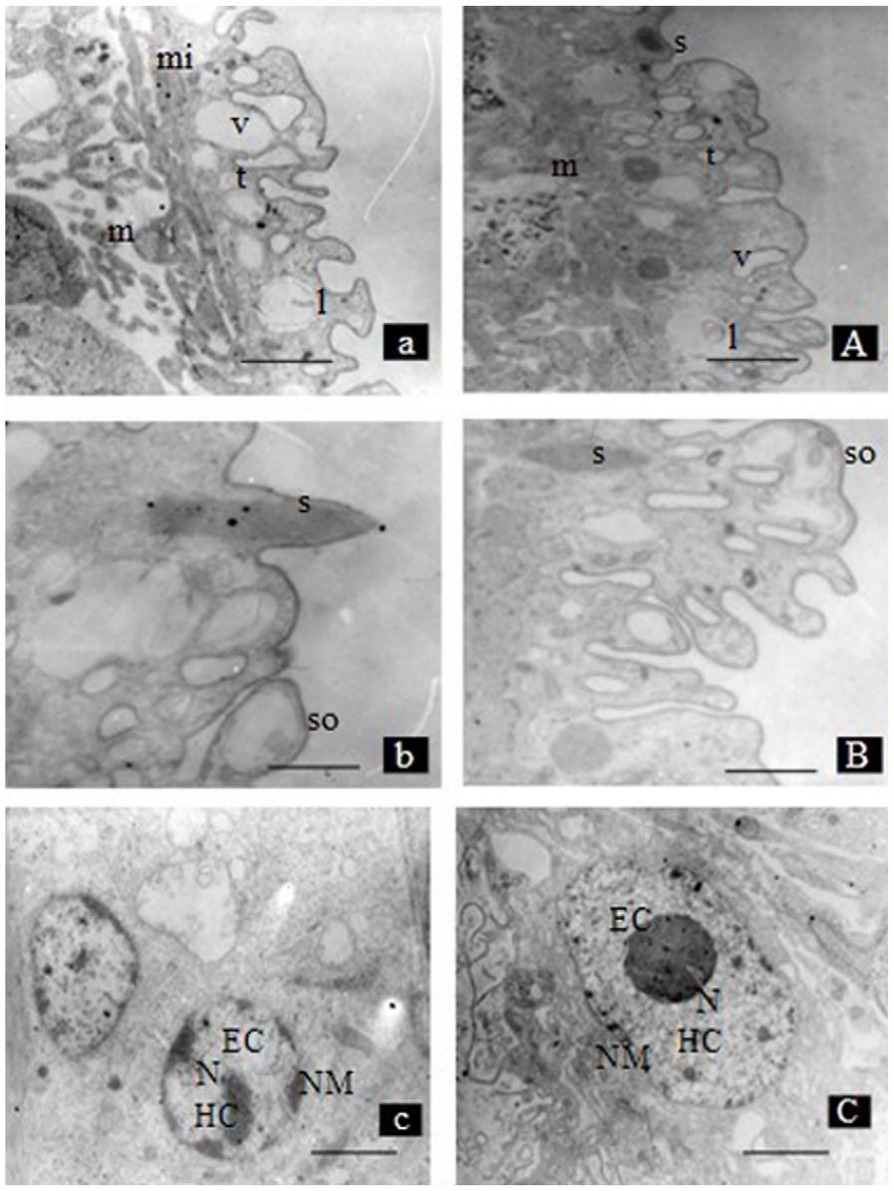
Ultrastructural differences in schistosomula from *Microtus fortis* (a, b, c) and BALB/c mice (A, B and C) evident by transmission electron microscopy (TEM): a/A Low magnification of the tegument region: muscle bundles (m); vesicles (v); tegument matrix (t); large bodies (l); mitochondria (mi). Bar  = 1 µm. b/B High magnification of the tegument region: surface spine (s); sensory organelle (so); Bar = 500 nm. c/C Ultrastructure of a single cell: euchromatin (Ec); heterochromatin (Hc); nuclear membrane (NM); Bar = 500 nm.

The ultrastructural differences of the schistosomula from the two different hosts are shown in Figure 2. Compared with schistosomula from BALB/c mice, the tegument of schistosomula from *M. fortis* had substantial focal lysis of the tegumental matrix, which resulted in the formation of vacuoles within the schistosomula. In addition, swelling and focal lysis of the underlying muscle bundles accompanied by degenerated mitochondria were evident (Figure 2a). The vacuoles seen in the sensory organelles distributed on the tegumental surface were caused by the focal swelling of the internal bilayer membrane and lysis of the tegumental matrix (Figure 2b). The schistosomula from *M. fortis* were also characterized by well recognized apoptotic features, such as reduced and condensed amounts of chromatin compressed against the nuclear envelope and aggregated into large dark, compact masses (Figure 2c). In contrast, the schistosomula from BALB/c mice were of normal appearance with a regular subcellular and nuclear structure (Figure 2A–C).

### Microarray analysis of differentially expressed genes in schistosomula

**Figure 3:**
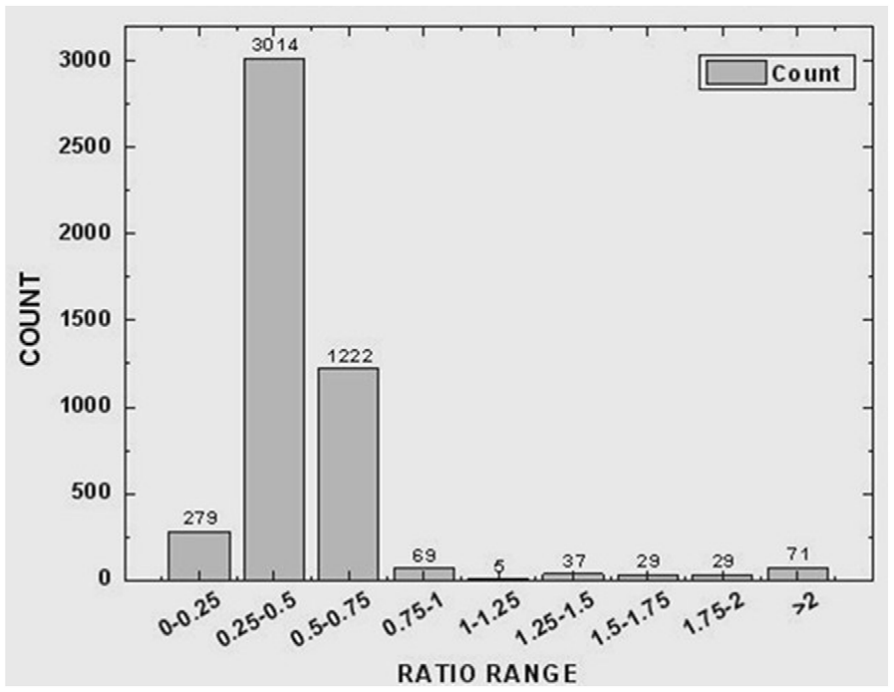
The frequency distribution of ratio values for the gene expression of the schistosomula from *Microtus fortis* compared with those from BALB/c mice.

All the gene expression data for the schistosomula from *M. fortis* was normalized with those from BALB/c mice. Statistical analysis revealed a large number of transcripts of schistosomula genes that were putatively related to the direct effect of the host species on the parasites. The frequency distribution of gene expression of the schistosomula is shown in Figure 3; differential expression was found in 3,364 genes, of which, 3,293 were down-regulated (≤2-fold) (Table S1), and 71 were up-regulated (≥2 fold) (Table S2) in schistosomula from *M. fortis* compared with those from BALB/c mice. Examples of genes showing significant differential expression in the schistosomula from *M. fortis* are presented in Tables 1 and 2.

**Figure 4:**
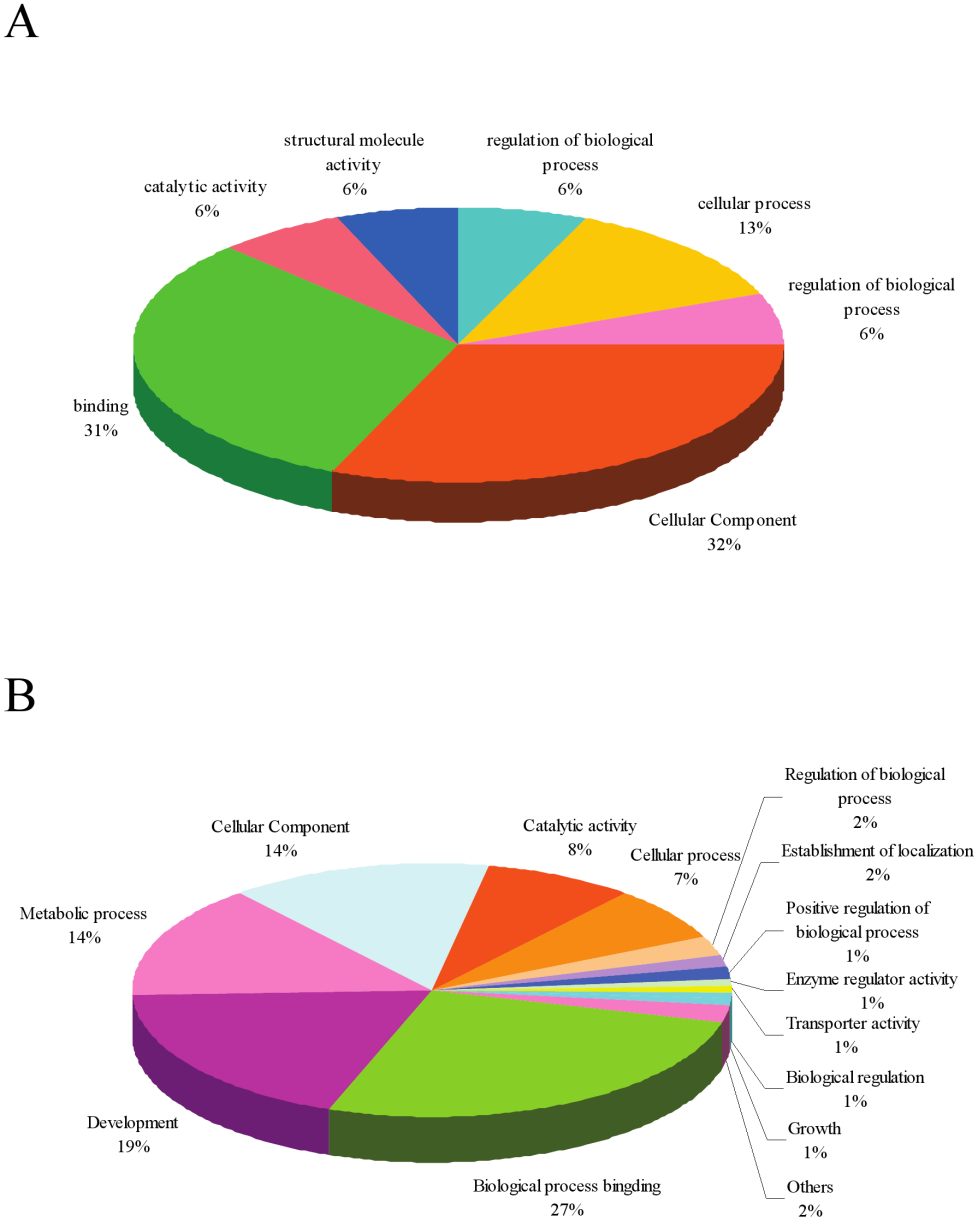
Pie chart showing the distribution of GOs for expressed genes in schistosomula from *Microtus fortis* A represents the category of the GOs for genes up regulated in worms from *Microtus fortis*. B represents the category of the GOs for genes down regulated in worms from *Microtus fortis*.

Of the 71 up-regulated genes, 16 had associated gene ontologies (GOs). Of these, seven were molecular function specific, five were cell component specific and four were biological process specific - these genes encode molecules that are involved mainly in binding, cellular processes and other molecular functions (Figure 4A). Of the 3,293 down-regulated genes, 1,427 had an associated GO. Of these, 527 were molecular function specific, 203 were cell component specific and 697 were biological process specific. As shown in Figure 4B, these genes encoded molecules that are involved mainly in biological processes (including development, metabolic processes, regulation of biological processes, transporter activity and growth) that have been highlighted owing to their possible molecular involvement in the development and growth of *S. japonicum*.

**Table 1:** Examples of up regulated expressed genes in schistosomula from *Microtus fortis* normalized with genes in those from BALB/c mouse.

Systematic Name	Gene ID	Protein Homology	Fold Ratio
Contig00705	SJCHGC01935	Far upstream element-binding protein 21	0.06
Contig05612	SJCHGC07194	Transportin-3	0.12
Contig07703	SJCHGC09378	Collagen alpha-2(I) chain	0.13
Contig07736	SJCHGC09413	Splicing factor, arginine/serine-rich 7	0.14
Contig04159	SJCHGC05650	Polypyrimidine tract-binding protein 1	0.15
Contig03880	SJCHGC05360	Proteasome inhibitor PI31 subunit	0.15
Contig05356	SJCHGC06922	Aryl hydrocarbon receptor repressor	0.15
Contig09151	SJCHGC10952	Mannitol-1-phosphate 5-dehydrogenase	0.15
Contig10888	SJCHGC12817	unknown	0.16
Contig05733	SJCHGC07317	Probable large tegument protein	0.16
Contig04261	SJCHGC05755	Phosphoribosyltransferase domain	0.16
Contig01742	SJCHGC03121	Peptidylprolyl isomerase domain	0.16
Contig00107	SJCHGC00584	CD63 antigen	0.16
Contig01794	SJCHGC03181	WD repeat and FYVE domain-containing protein 1	0.17
Contig10661	SJCHGC12582	Protein transport protein DSL1	0.17
Contig11091	SJCHGC13038	RNA pyrophosphohydrolase	0.17
Contig09718	SJCHGC11555	Cytochrome b	0.18
Contig03066	SJCHGC04511	Serum response factor-binding protein 1	0.18
Contig02162	SJCHGC03565	Serine hydroxymethyltransferase	0.18
Contig06989	SJCHGC08634	Transformation/transcription domain-associated protein	0.18
Contig03374	SJCHGC04836	AP1 subunit gamma-binding protein 1	0.18
Contig09945	SJCHGC11808	Protein transport protein	0.18
Contig07577	SJCHGC09244	Hybrid signal transduction histidine kinase L	0.18
Contig01784	SJCHGC03171	Pecanex-like protein 1	0.18
Contig13427	SJCHGC15542	Palmitoyltransferase	0.18
Contig11829	SJCHGC13830	DNA mismatch repair protein	0.18
Contig02940	SJCHGC04377	Thyroid receptor-interacting protein 11	0.19
Contig04390	SJCHGC05892	Serine/arginine repetitive matrix protein 2	0.19
Contig09120	SJCHGC10921	Frizzled-10-B	0.19
Contig05355	SJCHGC06921	Cathepsin Q	0.19
Contig06941	SJCHGC08583	Post-GPI attachment to proteins factor 2	0.19
Contig01706	SJCHGC03080	Dynein light chain 2	0.19
Contig01163	SJCHGC02477	Sugar phosphate exchanger 2	0.19

A full list of up regulated expressed genes in schistosomula from *Microtus fortis*, including systematic name, annotation on the microarray and protein homology, is shown in Table S6. 5 Fold change refers to expression relative to those from BALB/c mice.

**Table 2:** Examples of down-regulated expressed genes in schistosomula from *Microtus fortis* normalized with genes in those in schistosomula from BALB/c mice.

genes	qPCR analysis Fold ratio	microarray Fold ratio
CD63 antigen	0.28±0.06	0.16
Post-GPI attachment to proteins factor 2	0.12±0.08	0.19
magonashi	0.24±0.09	0.21
TGF-beta receptor binding protein	0.46±0.13	0.34
Fatty-acid amide hydrolase	0.35±0.17	0.4
Insulin receptor protein kinase	0.31±0.16	0.45
NADH-ubiquinone reductase	0.95±0.15	0.94
Tropomyosin	7.94±1.53	6.9
Granulins	56.7±5.42	44.3

A full list of up regulated expressed genes in schistosomula from *Microtus fortis*, including systematic name, annotation on the microarray and protein homology, is shown in Table S6. 0.2 Fold Ratio refers to expression relative to schistosomula from BALB/c mice.

GO analysis also revealed that some of the differentially expressed genes have important biological functions. Among these, genes up-regulated in schistosomula from *M. fortis* included those encoding proliferation-related granulin and tropomyosin. The genes down-regulated in schistosomula from *M. fortis* included those encoding metabolism-related insulin-2 (Contig06300), insulin receptor protein kinase (Contig07581), long-chain fatty acid transport protein(Contig04819), fatty acid amide hydrolase (Contig07100), fatty acid-binding protein (Contig00065), fatty acid desaturase (Contig04324), signal transduction-related molecules, TGF pathway-related molecules, eukaryotic translation initiation factor 3 (Contig04576) and TGF-β1 (Contig10153), Wnt pathway-related molecules, Wnt inhibitory factor 1 (Contig05142), development-related MAP 2 (Contig04100) and mago nashi (Contig01249).

Of the differentially expressed genes, 270 had specific KEGG pathway annotations. Of these, 269 were down-regulated and were involved mainly in oxidative phosphorylation, glycolysis and/or gluconeogenesis, fatty acid biosynthesis, MAPK signaling pathways, insulin signaling pathways and apoptosis (Table S3).

### qPCR validation

Nine genes with different biological functions were selected for qPCR to validate the microarray transcription results. The primer sequences for the qPCR are listed in Table S4. NADH-ubiquinone reductase was used to normalize the expression difference in schistosomula from *M. fortis*. In addition, the expression of the nine selected genes in schistosomula from *M. fortis* was normalized by using the expression of genes in schistosomula from BALB/c mice (the relative gene expression of those from BALB/c mice were used to standardize the ratio) [15]. The relative differential gene expression obtained by microarray analysis and by quantitative PCR was similar for the majority of data points for the 9 genes assessed (Table 3). There was a significant correlation (alpha  = 0.05) of 0.8833 between the two data sets (Spearman's Rho, *P* = 0.0031, *n* = 18).

**Table 3:** Results of real-time qPCR confirmation of gene microarray data.

	AnnexinV^+^PI^−^(%)	AnnexinV^+^PI^+^ (%)
Cells of schistosomula from BALB/c mice	0.47±0.26	0.25±0.12
Cells of schistosomula from *Microtus fortis*	39.23±2.95**	3.81±0.42**

### Analysis of the apoptosis-associated genes with qPCR

**Figure 5:**
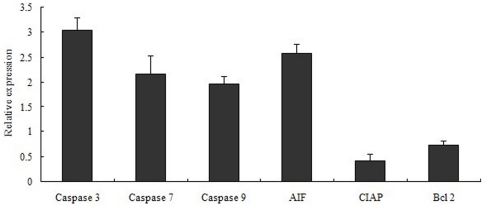
Relative expression of six genes in schistosomula from *Microtus fortis* compared with those from BALB/c mice by using qPCR. The data show the mean and standard error derived from triplicate experiments.

The primer sequences used for the qPCR are listed in Table S5. Analyses of the apoptosis-associated genes in schistosomula from *M. fortis* revealed that some apoptosis-inducing genes and forward-regulating genes such as those encoding *Sj* caspase 3, *Sj* caspase 7, *Sj* caspase 9 and AIF, were up-regulated 3, 2, 2, 2.5 fold, respectively. The apoptosis-inhibiting genes and down-regulated genes, such as those encoding molecules involved in apoptosis antagonism - for example, CIAP and Bcl-2 - were down regulated by 50% and 25%, respectively, in schistosomula from *M. fortis* compared with those from BALB/c mice (Figure 5).

### Caspase activity analysis

**Figure 6:**
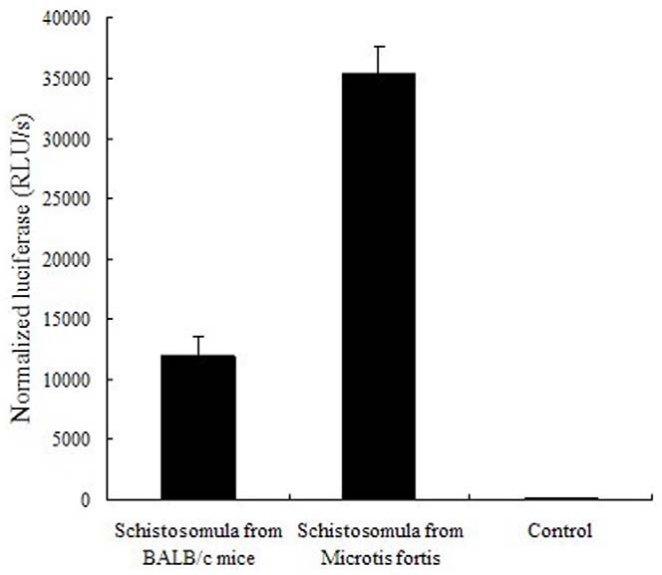
Caspase activity analysis. The caspase activity in schistosomula from BALB/c mice and *Microtus fortis* show the mean and standard error derived from triplicate experiments.

Caspase activity in the schistosomula from BALB/c mice and from *M. fortis* was determined using a Glo™ 3/7 Assay kit (Figure 6). Caspase activity was higher in the schistosomula from *M. fortis* than in those from BALB/c mice (*p*<0.01).

### Cell proliferation assays and apoptosis analysis

**Figure 7:**
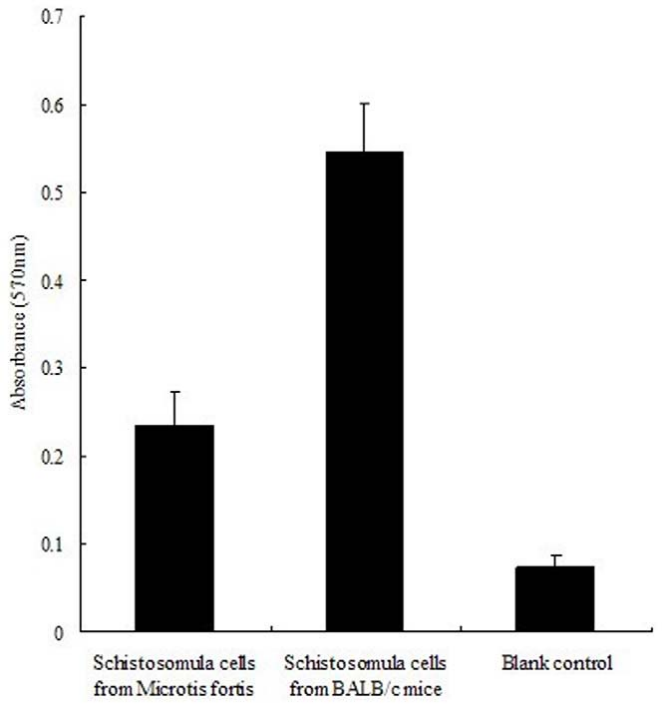
Proliferation of schistosomula cells obtained with schistosomula taken from BALB/c mice and *Microtus fortis* using the MTT assay. Cultures containing no cells comprised the blank control.

MTT assays were performed to determine the proliferation of schistosomula cells from the two host species. As shown in Figure 7, the proliferation rate of the schistosomula cells from BALB/c mice was significantly higher than those from *M. fortis* (*p*<0.01), and the control was lower than those of either sample of schistosomula (*p*<0.01)

**Figure 8:**
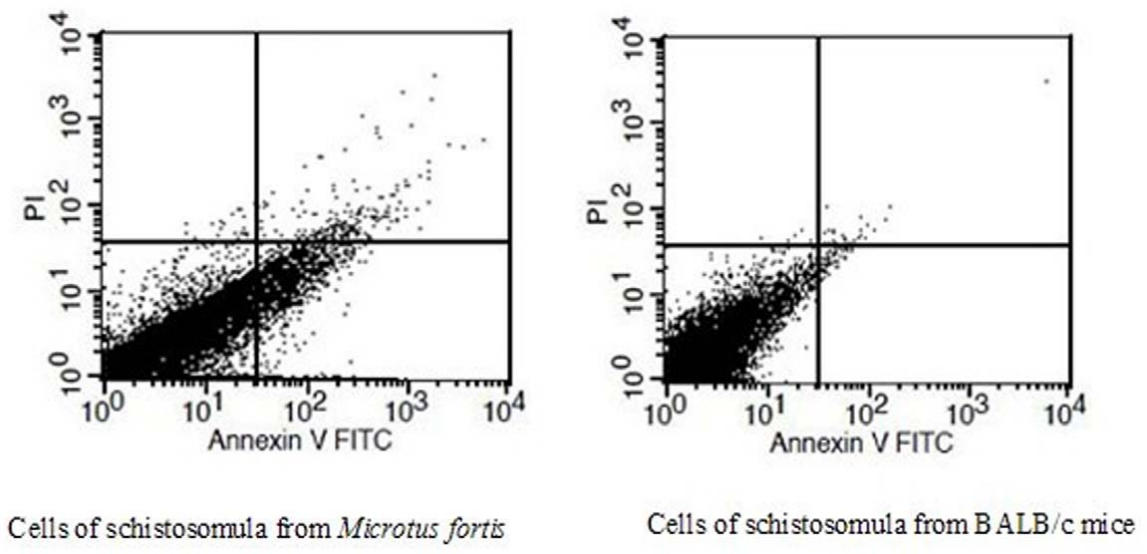
Apoptotic and necrotic cells of schistosomula taken from from BALB/c mice and *Microtus fortis* determined by Annexin V-FITC/PI analysis. The lower left quadrants refer to intact cells (AnnexinV^−^PI^−^); the lower right quadrants show early apoptotic cells (AnnexinV^+^PI^−^); the upper right quadrants refer to late apoptotic and/or necrotic cells (AnnexinV^+^PI^+^). The figures are representative of three independent experiments.

The Annexin V-FITC/PI apoptosis detection kit was applied to determine quantitatively the percentage of apoptotic schistosomula cells by Annexin V-propidium iodide staining. The quantification of phosphatidylserine residues redistributed from the inner to the outer leaflet of the cell membrane is an early event in apoptosis. Three populations of cells could be distinguished including viable (no staining), early apoptotic (AnnexinV^+^PI^−^) and late apoptotic/necrotic (AnnexinV^+^PI^+^) cells (Figure 8). The results showed that the percentage of early apoptotic and late apoptotic/necrotic cells in schistosomula from *M. fortis* was significantly higher than in those from BALB/c mice (*p*<0.01) (Table 4), indicating that apoptosis was more prevalent in schistosomula cells from the former host species.

**Table 4:** Apoptosis and necrosis in cells of schistosomula taken from the two host species determined by Annexin V-FITC/PI analysis (Mean ± SD; N = 3).

	Fluo-12UTP^+^PI^−^(%)	Fluo-12UTP^+^PI^+^ (%)
Cells of schistosomula from BALB/c mice	0.26±0.04	0.52±0.14
Cells of schistosomula from *Microtus fortis*	28.80±4.76**	1.75±0.35**

**P<0.01 (the cells of schistosomula taken from *Microtus fortis* compared with those taken from BALB/c mice).

### TUNEL analysis of schistosomula cells

**Figure 9:**
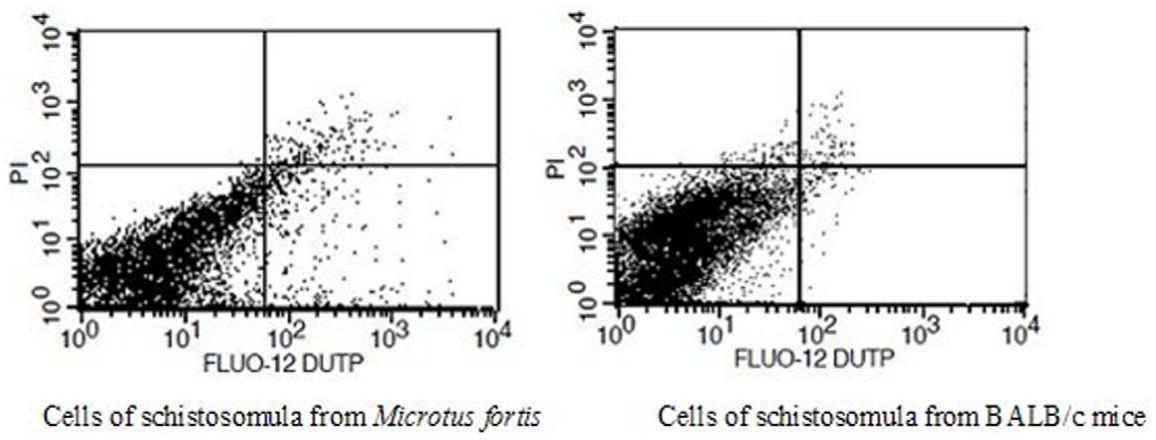
Tunnel analysis of cells of schistosomula taken from BALB/c mice (right panel) and *Microtus fortis* (left panel). The lower left quadrants refer to intact cells (FLUO-12UTP^−^PI^−^); the lower right quadrants represent apoptotic cells (FLUO-12UTP^+^PI^−^); the upper right quadrants refer to necrotic cells (FLUO-12UTP^+^PI^+^). The data shown are representative of three independent experiments.

The Fluo-12UTP/PI apoptosis detection kit was used to determine quantitatively the percentage of apoptotic schistosomula cells. The population of viable cells (no staining) could be readily distinguished from those of apoptotic (Fluo-12UTP^+^PI^−^) and late apoptotic and/or necrotic (Fluo-12UTP^+^PI^+^) cells (Figure 9).The results showed that the percentage of apoptotic and late apoptotic/necrotic cells in schistosomula from *M. fortis* was significantly higher than in those from BALB/c mice (*p*<0.01) (Table 5).

**Table 5:** Apoptosis and necrosis in cells of schistosomula taken from the two host species determined by Fluo-12UT/PI analysis (Mean ± SD; N = 3).

Systematic Name	Gene ID	Protein Homology	Fold change
Contig08554	SJCHGC10874	E3 ubiquitin-protein ligase ubr11	88.2
Contig12492	SJCHGC08077	Granulins	44.3
Contig09693	SJCHGC13643	unknown	18.2
Contig12392	SJCHGC16303	Cell surface receptor daf-4	14.7
Contig00716	SJCHGC02637	Pericentriolar material 1 protein	9.5
Contig00449	SJCHGC09772	Protein TAR1	8.2
Contig01305	SJCHGC01953	Dynein heavy chain (Fragment)	8.0
Contig07698	SJCHGC14859	Zinc finger RNA-binding protein	7.7
Contig14121	SJCHGC14422	unknown	7.5
Contig11651	SJCHGC11527	Tropomyosin	6.9
Contig13194	SJCHGC02429	Putative BTB/POZ domain-containing protein	6.9
Contig06456	SJCHGC14529	ATP-dependent RNA helicase DBP10	5.9
Contig09076	SJCHGC10331	Probable serine/threonine-protein kinase	5.0

**P<0.01 (the cells of schistosomula taken from *Microtus fortis* compared with those taken from BALB/c mice).

## Discussion

A number of previous studies have shown that whereas the BALB/c mouse is a susceptible host for *S. japonicum*, *M. fortis* is non-permissive. The maturation rate of *S. japonicum* in BALB/c mice is ∼70%, but few if any parasites are present in *M. fortis* 15 days post-infection as the schistosome fails to complete its life cycle in this rodent model. Consequently, *M. fortis* is the only known mammalian host in which *S. japonicum* that does not induce pathology and, as such, it has been used as an animal model to investigate the mechanisms which lead to the disease of schistosomiasis. In recent years, schistosomiasis control has relied mainly on the chemotherapeutic drug praziquantel, but frequent re-infection of humans and animals occurs in endemic areas following treatment, and there are increasing concerns about possible drug resistance in the future [19]. Therefore, screening for additional drug targets and the identification of new vaccine candidates is required for the successful future control of schistosomiasis [20]. Comparing gene expression differences between schistosomula from susceptible BALB/c mice and non-permissive *M. fortis* will be helpful for identifying molecules that might be important for schistosome survival and development. Such a comparison will also aid in our understanding of the developmental and growth mechanisms of schistosomes in the mammalian host.

In the present study, the morphology of 10-day-old schistosomula collected from BALB/c mice and *M. fortis* were compared using SEM, TEM and light microscopy, and clear differences were evident. Schistosomula from *M. fortis* were characterized by a withered blebby appearance, tegument sloughing, and the formation of vacuoles in the tegument; in addition, some structures, such as muscle bundles and sensory organelles, were damaged compared with the schistosomula from BALB/c mice. The study also revealed that schistosomula from *M. fortis* appeared to have retarded growth and other changes such as swelling and focal lysis of the underlying muscle bundles. These effects on the parasite may represent one point in a series of events which culminate in the killing of *S. japonicum* 5 days later at day 15 post infection. However, what is not clear is the nature of the molecular mechanisms that lead to these obvious morphological/phenotypical differences.

To investigate possible mechanisms involved in the retardation of parasites developing within *M. fortis*, a schistosome transcriptome-wide oligonucleotide microarray was used to compare and analyze the global gene expression profile in schistosomula from *M. fortis* and BALB/c mice. Genes encoding granulin and tropomyosin were more highly expressed in the schistosomula from *M. fortis*. The function of granulin in schistosomes has not been determined but it is present in the excretory/secretory (ES) products of another trematode, *Opisthorchis viverrini,* as a secreted growth factor that induces proliferation of host cells and has been implicated as having an important role in the development of cholangiocarcinoma [21]. The over-expression of the gene encoding granulin in schistosomula from *M. fortis* suggests that granulin also has an important role in the proliferation and growth of *S. japonicum*; granulin could be a target for host immune attack via antibody-dependent cellular cytotoxicity in *M. fortis*. Tropomyosin is a major regulatory molecule of the contractile system in muscle as well as an important component of the cytoskeleton in non muscle cells, and it has been considered as highly antigenic and a major anti-schistosome vaccine candidate [22]. By contrast, some of the down-regulated genes in the schistosomula obtained from *M. fortis* are associated with development, metabolic, signal transduction and growth processes and likely have important biological functions in the survival and development of *S. japonicum*. Additionally, some of these down-regulated genes are also involved in metabolic processes including carbohydrate, lipid and amino acid metabolism.

Recent findings of Jiang *et al.* [20] indicated that the expression of developmental genes such as *Thra, Thrsp, Hsd11b1 and Igf1* was significantly lower in the lungs or liver of *M. fortis* infected with *S. japonicum,* compared with infected BALB/c mice. Therefore, the down-regulation of these development-associated genes in *M. fortis* might be a reason for the failure of schistosomula to grow and develop in this host.

The recent annotation of the genome of *S. japonicum* revealed that pathways for fatty acid biosynthesis and metabolism were incomplete and that some enzymes involved in fatty acid degradation and synthesis were important in lipid metabolism in this schistosome [8]. The reduced expression of genes associated with fatty acid and steroid biosynthesis indicated that fatty acid metabolism was suppressed in schistosomula obtained from *M. fortis*. In addition, some molecules involved in small-molecule metabolism, such as the insulin signaling pathway and Vitamin B6 metabolism, were found to be down regulated in the schistosomula from *M.* fortis. Insulin has an important role in schistosome development and could affect the sexual differentiation and fecundity of female schistosomes by activating the MAPK sub-pathway [23], [24]. The results detailed in Table S3 suggest that insulin receptor/host ligand binding by schistosomula from *M. fortis* host is limited, which in turn may have influenced their development. Genes encoding some of the molecules involved in the Wnt and TGF-β signal pathways were also found to be significantly down-regulated in schistosomula from *M. fortis*. The Wnt extracellular signaling pathway and the TGF-β superfamily are two important signaling pathways in the development of many species. The Wnt extracellular signaling pathway is an evolutionarily conserved signal transduction pathway of animal development [25]. Members of the TGF-β superfamily are secreted polypeptide growth factors that have important and diverse roles in cellular growth, differentiation, extracellular matrix formation and immuno-suppression [26]. The results reported here suggest that gene expression of schistosomula is controlled and regulated by a series of precise signal transduction pathways, and the reduced expression of genes encoding molecules involved in insulin, Wnt, TGF-β and other signaling pathways might be one of the important factors reducing the viability of schistosomula in *M. fortis*. The results also suggest that these genes, and the molecules they encode, are essential for the survival, growth and development of *S. japonicum* in viable hosts and, therefore, they could represent good targets for drug or vaccine development for controlling or preventing schistosomiasis in permissive hosts, including humans and the the numerous animal reservoirs of schistosomiasis japonica.

Apoptosis has been reported in most multicellular organisms, including nematodes, insects, amphibians and mammals [7]. Genomic studies indicate that several molecules are involved in the apoptotic pathway in *S. japonicum* and that apoptosis might be an important event in the interplay between parasites and their hosts [15], [27], [28]. There are very limited descriptions of ultrastructural changes in *S. japonicum* undergoing apoptosis but here, typical apoptotic features, such as morphological changes and condensed amounts of chromatin, were characteristic of schistosomula obtained from *M. fortis*.

Several methods have been established to evaluate cell vitality and apoptosis at the individual cell level. To evaluate cell vitality and apoptosis status in schistosomula, single cell preparations of schistosomula were obtained by trypsin digestion [29]. The cell proliferation assays revealed that cells of schistosomula harvested from BALB/c mice had greater active proliferation ability compared with those from *M. fortis*. Corresponding with the ultrastructure observations of apoptosis in schistosomula from the two different host species, 39.23% and 3.81% (via Annexin V-FITC/PI analysis) and 28.80% and 1.75% (via Fluo-12UTP/PI analysis) of schistosomula cells from *M. fortis* were characterized as either early-apoptosis cells or late-apoptosis cells, respectively. By contrast, only 0.47% and 0.25% (via Annexin V-FITC/PI analysis) and 0.26% and 0.52% (via Fluo-12UTP/PI analysis) schistosomula cells from BALB/c mice were classified as either early-apoptosis cells or late-apoptosis cells, respectively.

Whereas, Annexin V-FITC analysis is based on the redistribution of phosphatidylserine residues from the inner to the outer leaflet of the cell membrane in early apoptosis, TUNEL analysis is based on measurements of nuclear DNA fragmentation. The detecting of the redistribution of phosphatidylserine residues from the inner to the outer leaflet of the cell membrane can be detected in both early apoptosis and apoptosis cell, the measurements of nuclear DNA fragmentation can only be detected in the apoptosis cells. Changes in nuclear DNA fragmentation and the redistribution of phosphatidylserine residues are both specific to the process of apoptosis. The difference in the proportion of apoptotic schistosomula cells measured from *M. fortis* by these different methods could reflect the difference in sensitivity between them. The apoptosis analysis directly indicated that apoptosis occurs in living schistosomula of *S. japonicum* and further confirms the existence and biological function of apoptosis genes in the schistosome genome.

The caspases are a subfamily of cysteine proteases that have key regulatory roles in the apoptotic process; and caspase 3, 7 and 9 have been characterized as key factors in apoptosis execution in many organisms [7]. In the current study, caspase 3 and 7 activities were measured using a Glo™ 3/7 Assay kit, and the expression of their genes were determined by qPCR analysis. The mRNA level expression of these two genes determined by the micraoarray analysis was not as highly regulated as was shown by the qPCR analysis, which is is a more sensitive technique and the results more reliable [30]. The results of the qPCR analysis demonstrated that the level of gene expression and caspase 3 and 7 activities in the schistosomula from *M. fortis* were significantly higher than in those from BALB/c mice. In the mammalian apoptosis pathway, some molecules, such as AIF, and other apoptosis-inhibiting molecules such as CIAP, Bcl-2 and inhibitor of apoptosis (IAP), are a family of apoptosis-related proteins that regulate caspases 3, 7 and/or 9. The expression of the upstream forward-regulating gene encoding AIF was significantly up-regulated, but the downstream genes encoding CIAP, Bcl-2 and IAP were significantly down-regulated in schistosomula from *M. fortis*. Peng *et al.* reported that the eukaryotic expression plasmids of *S. japonicum* IAP can reduce caspase activity in 293T cells [9]. Skin-stage schistosomula of *S. mansoni* were confirmed to secrete molecules that are pro-apoptotic for skin T lymphocytes [28]. Jiang *et al.* reported that the analysis of the apoptosis-inducing genes in infected *M. fortis* were up-regulated compared with those in uninfected hosts; the authors suggested that the role of apoptosis receptors and related proteins in *M. fortis* might be to initiate apoptosis in the schistosome [20]. The apoptosis intrinsic pathway when specifically triggered by external signals begins an energy-dependent cascade of molecular events which eventually cause the apoptosis phenomenon [7]. However, few molecules had been characterized in the schistosome apoptosis pathway. Based on the current results, including the importance of signal molecules, such as caspase 3, caspase 9, caspase 7 and IAP, in the apoptosis pathway, we can conclude that the intrinsic apoptosis pathway may be one of the main pathways triggered in schistosomula from *M.fortis* (Figure S1). Thus, it is possible that apoptosis-related molecules in schistosomes may impact on and interact with apoptosis regulation in the animal host, and that this is a critically important process affecting the survival and development of schistosomula in *M. fortis.*

In summary, the current study has shown that the morphologies of *S. japonicum* schistosomula collected from susceptible BALB/c mice and non-permissive *M. fortis* were substantially different. Further investigation revealed that the apparent growth retardation and pathological changes observed in schistosomula from *M. fortis* might be the result of apoptosis and the differential expression of some apoptotic, developmental, metabolic and signal transduction-related genes that were significantly influenced by the different internal environments of the two host species. These, in turn, would adversely affect the survival and development of *S. japonicum* in the non-permissive *M. fortis,* and lead ultimately to the death of the migrating schistosomula.

## Supporting Information

Table S1 A full list of genes, including systematic name, Gene ID and protein homology, up regulated in schistosomula from *M.fortis* 2 Fold change refers to expression relative to those of schistosomula from BALB/c mice.
(XLS)

Table S2 A full list of genes, including systematic name, Gene ID and protein homology, up regulated in schistosomula from *M.fortis* 0.5 Fold ratio refers to expression relative to those of schistosomula from BALB/c mice.
(XLS)

Table S3 KEGG analysis of differentially expressed genes in the schistosomula from *Microtus fortis* compared with those from BALB/c mice.
(XLS)

Table S4 Information on the primers used for validation of the microarray data by quantitative qPCR analysis.
(XLS)

Table S5 Information on the primers used for validation of the microarray data by quantitative qPCR analysis.
(XLS)

Table S6 A full list of genes, including systematic name, Gene ID and protein homology significantly up and down regulated in schistosomula from *Microtus fortis.*
(XLS)

Figure S1 The apoptosis signal pathway in *Homo* and *S. japonicum* (from the *S. japonicum* genome project database;http://chgc.sh.cn/japonicum/).
(DOC)
