# Supplementary material for: Apoptosis Governs the Elimination of Schistosoma japonicum from the Non-Permissive Host Microtus fortis
Source: PLoS One. 2011 Jun 22;6(6):e21109. doi: 10.1371/journal.pone.0021109 (PMC3120819; doi:10.1371/journal.pone.0021109)
Supplement: Figure S1 — The apoptosis signal pathway in Homo and S. japonicum (from the S. japonicum genome project database;http://chgc.sh.cn/japonicum/). (DOC) [file pone.0021109.s007.doc]

a


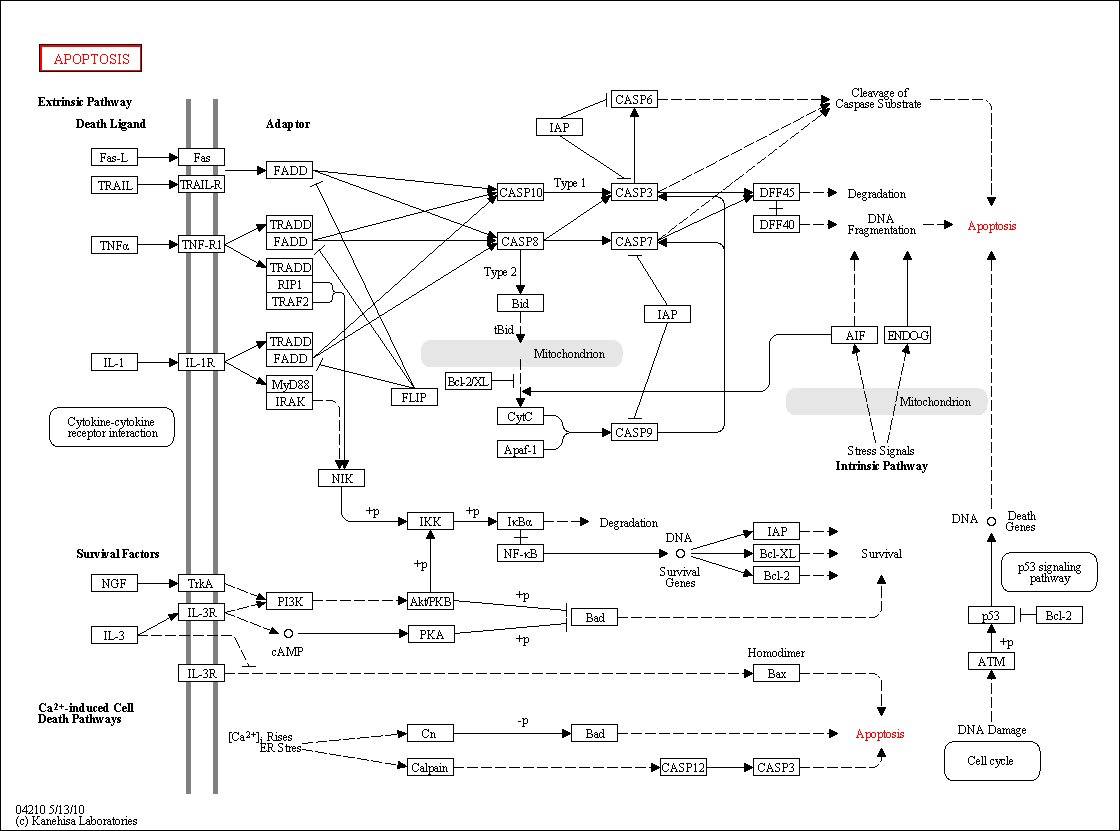


b


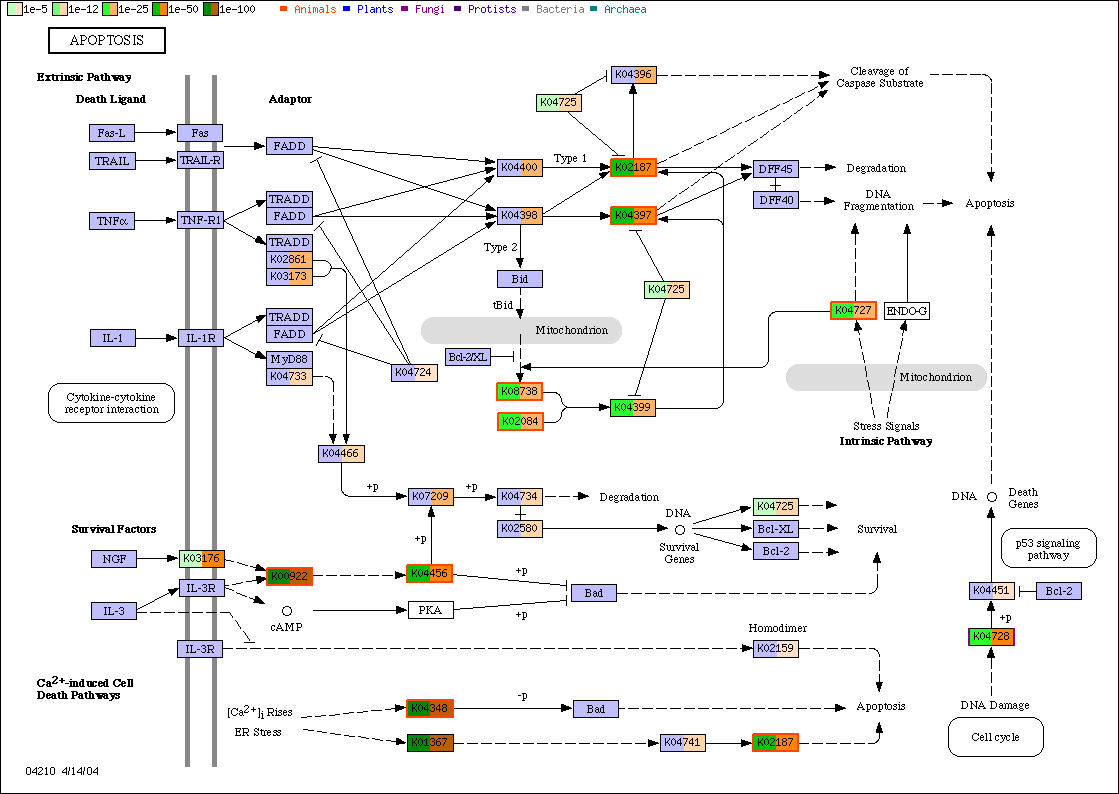


Figure S2.

the apoptosis signal pathway in human from <http://www.genome.jp/kegg/pathway.html>(a)and the apoptosis signal pathway of schistosome in S. japonicum genome project database (http://chgc.sh.cn/japonicum/) (b).
